# Gastrointestinal Imaging Findings in the Era of COVID-19: A Pictorial Review

**DOI:** 10.3390/medicina59071332

**Published:** 2023-07-19

**Authors:** Xanthippi Mavropoulou, Elisavet Psoma, Angeliki Papachristodoulou, Nikoletta Pyrrou, Ekaterini Spanou, Maria Alexandratou, Maria Sidiropoulou, Anastasia Theocharidou, Vasileios Rafailidis, Theofilos Chrysanthidis, Panos Prassopoulos

**Affiliations:** 1 Department of Clinical Radiology, AHEPA University Hospital of Thessaloniki, Aristotle University of Thessaloniki, 54634 Thessaloniki, Greecebillraf@hotmail.com (V.R.);; 2 Infectious Diseases Division, First Internal Medicine Department, School of Medicine, Aristotle University of Thessaloniki, 54634 Thessaloniki, Greece

**Keywords:** COVID-19, abdominal, gastrointestinal, CT/CTA, pancreatitis, colitis cholecystitis, pancreatitis, bleeding

## Abstract

The potentially fatal COVID-19 pandemic has been associated with a largespectrum of clinical presentations. Beyond the classical pulmonary manifestations, gastrointestinal tract-related symptoms suchas nausea, diarrhea, abdominal distention and pain have been observed in patients, as a consequence of the binding of SARS-CoV-19 to Angiotensin-converting Enzyme 2 (ACE2) receptors in the gastrointestinal (GI) tract. The early recognition ofspecific imaging features, including hepatobiliary involvement, pancreatic involvement, development of solid organ infarcts, ischemic bowel changes and vascular occlusion, plays a key role through the course of the disease. Also, suspicious symptoms, especially in critically ill patients with clinical and biochemical markers of hypovolemia, necessitate timely imaging for bleeding complications. The aim of this pictorial review is to illustrate the spectrum of the GIimaging findings in patients with COVID-19. Awareness of diagnostic imaging hallmarks is crucial to optimize the management of these patients.

## 1. Introduction

Since December 2019, the world has observed the appearance and spread of SARS-CoV-19, a novel coronavirus that causes a severe respiratory syndrome (COVID-19) and its disastrous impact on global health [1,2]. COVID-19 was declared as a pandemic on March 11, 2020 by the World Health Organization (WHO), and since then millions of people worldwide have been affected. As of May 2023, more than six million deaths have been confirmed.

Although in the majority of cases, the disease manifests with respiratory symptoms such as cough, fever and dyspnea, its extra-pulmonary manifestations are being increasingly acknowledged [3] Specifically, the incidence of GI manifestations such as abdominal pain, nausea or diarrhea has ranged from 12% to as much as 61% in patients with COVID-19. Moreover, some patients might present only with GI symptoms, hindering diagnosis. Furthermore, it has been noted that patients presenting with GI symptoms tend to progress more to a severe form of disease with poor outcomes [4,5,6]. As it is well documented that SARS-CoV-19 uses ACE-2 receptors to enter human cells, this receptor is found to be expressed not only in pulmonary epithelial cells but also in gastrointestinal and hepatobiliary cells, explaining the GI involvement [7,8]. Despite the broad recognition of respiratory imaging findings, until now few studies have been published about abdominal radiological presentation of patients with COVID-19 [9,10].

Taking into consideration the high possibility of GI tract involvement, it is crucial for radiologists to be aware of the variety of abdominal imaging findings in patients with COVID-19, as early recognition can aid the diagnosis in patients with nonspecific, atypical symptoms.

## 2. Materials and Methods

The aim of this study is to share our experience and present the imaging findings of GI manifestations that can be noted in patients with COVID 19.

The authors executed a retrospective study from our hospital archives starting from February 2020, when the first COVID-19 patient was admitted in our hospital, until 31 December of 2022. Six of the authors searched the institutional databases of COVID-19 patients who underwent abdominal CT. Confirmed COVID-19 patients who presented with abdominal signs and symptoms were included in the study. A dedicated 16-slice SIEMENS EMOTION CT scanner was used for confirmed COVID-19 cases. The final study population was 84 patients. Simultaneously, a search of the published literature was conducted on PubMed using suitable key words (e.g., COVID-19, abdominal manifestations, CT, imaging, and gastrointestinal).

### 2.1. Colon Manifestations

The bowel is the most commonly involved abdominal organ in COVID-19 patients. Frequent abdominal imaging features include intestinal imaging findings (24%), including colorectal (5%) and small bowel thickening (12%), intestinal distension (18%), pneumatosis and intestinal perforation [11]. Bhayana R. et al. observed [9] similar bowel-wall abnormalities in 31% of CT images in patients in the Intensive Care Unit (ICU). Common indications for CT evaluation include abdominal pain or distention, hematochezia and nausea [3]. It is important to note that the presence of bowel abnormalities predict worse prognosis and increased clinical severity.

ACE 2 receptors are located in gastrointestinal cells (enterocytes and vascular cells) and can interpret the bowel manifestations of the virus, specifically the inflammatory bowel manifestations and ischemic bowel alterations. This is supported by the detection of SARS-CoV-19 virus in feces samples [4,12]. Infection of the GI tract epithelial cells is followed by an inflammatory response and a cytokine release.

**Figure 1:**
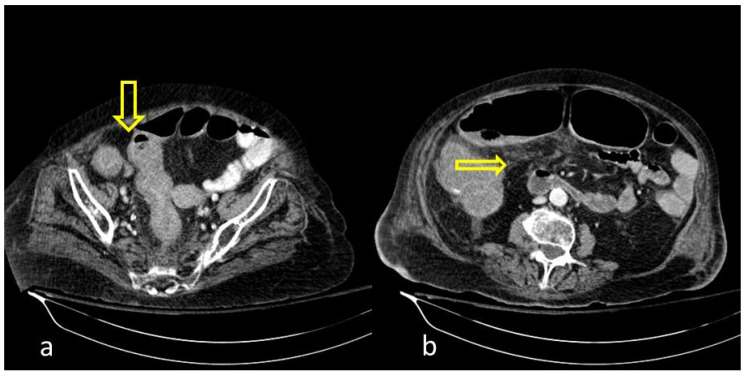
COVID-19 (+) hospitalized 75-year-old male patient with abdominal pain and diarrhea. Abdominal Contrast Enhanced CT (CECT) depicted bowel wall thickening ((**a**), thick arrow) and mesenteric fat stranding ((**b**), thin arrow). The imaging findings are indicative of inflammatory colitis.

**Figure 2:**
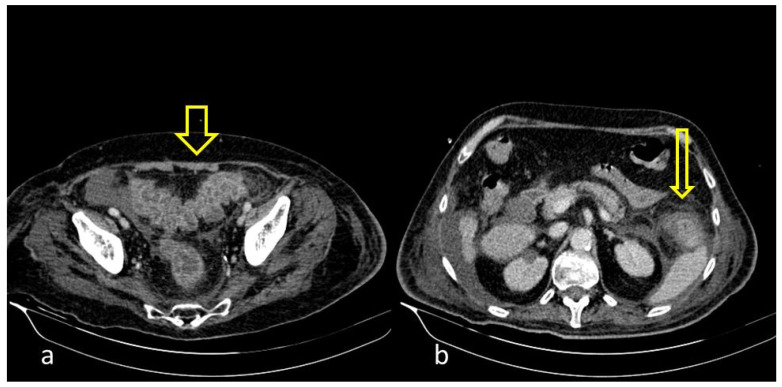
COVID-19 (+) 68-year-old patient with a long hospital stay was subjected to an abdominal CECT for acute abdominal pain, which showed colonic wall thickening ((**a**), thick arrow) and adjacent fat stranding ((**b**), thin arrow).

**Figure 3:**
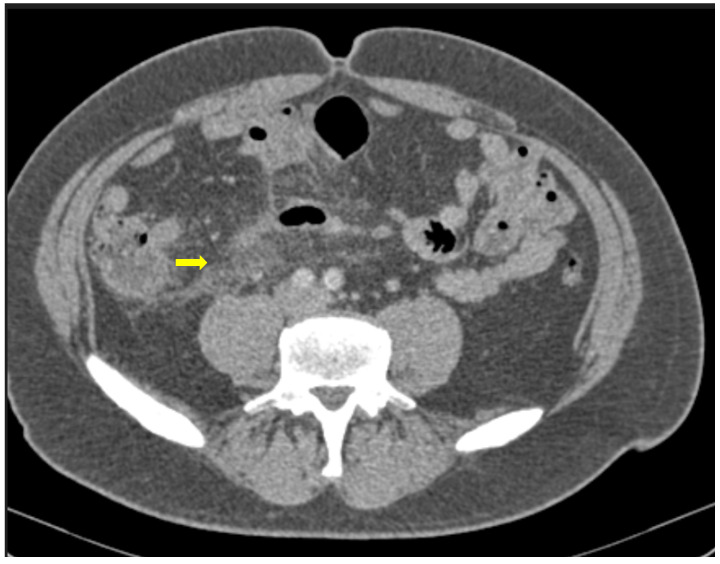
55-year-old male patient with COVID-19 pneumonia with right lower quadrant pain. CT on admission demonstrating a thickened appendix and proximal fat stranding (yellow arrow), indicating acute appendicitis.

**Figure 4:**
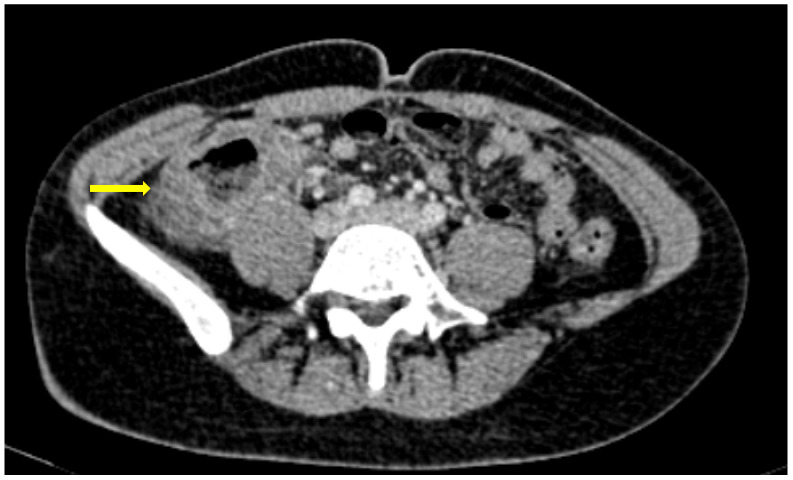
A 48-year-old female patient with COVID-19 pneumonia and no other comorbidities. CT scan depicted a thickened cecum with proximal fat stranding (yellow arrow).

A large bowel infection usually appears with diffuse circumferential and enhancing wall thickening (hyperenhancement) that can involve one or more segments of the colon (Figure 1 and Figure 2). Pericolic fluid or perintestinal fat stranding is common while pericolic lymphadenopathy is not (Figure 3 and Figure 4). If we suspect COVID-19-related colitis, clinical correlation is needed, and the detection of the virus in stools can establish the diagnosis.

It is very important to suspect and diagnose patients presenting acute mesenteric ischemia (AMI) or perforation of the bowel [13]. It is well documented that critically ill COVID-19 patients are at risk for thrombosis or bleeding [14].

AMI, is associated with severe symptoms, a worsening systemic status and high morbidity and mortality. Imaging has a crucial role in its detection and is the cornerstone of diagnosis [15]. An ultrasound is nonspecific with low sensitivity but may reveal decreased peristalsis and intraluminal content that indicate stasis.

CT angiography remains the pillar for detection of signs of ischemia [16]. Filling defects that represent emboli or thrombi within the lumen of the abdominal aorta and its branches are major findings along with hypoenhancement of the mesenteric vascular arcade and decreased contrast enhancement of the bowel wall that indicates hypoperfusion. In some cases, a target appearance of the bowel wall, representing mucosal hyperemia with adjacent mural edema, can be depicted in ischemic colitis. The affected segment appears with thickened bowel walls, and it may be pictured as fluid-filled due to disruption of peristalsis [17]. Additional findings are porto-mesenteric venous gas, pneumatosis intestinalis and pneumoperitoneum [18,19]. In a later phase, the bowel wall is thinned due to loss of normal tone.

### 2.2. Small Intestine Manifestations

When COVID-19 affects the small intestine, it can cause a range of symptoms, including diarrhea, abdominal pain and vomiting, in both adults and children [20].

One study published in the Journal of Medical Virology examined the fecal samples of COVID-19 patients and found that the virus was present in the samples of 23 out of 29 patients, indicating that SARS-CoV-19 can be transmitted via the fecal–oral route. Additionally, the virus was present in higher amounts in patients with gastrointestinal symptoms, such as diarrhea and nausea [21].

Another study found that nearly half of COVID-19 patients had gastrointestinal symptoms, such as diarrhea, nausea and vomiting. Those patients experienced a longer duration of illness compared to those without digestive symptoms [22].

The exact mechanisms by which COVID-19 affects the small intestine are still not fully understood, but it is thought that the virus may directly infect the intestinal cells or that it may cause an inflammatory response that affects the gut. Additionally, some patients may experience a dysregulated immune response, which can also contribute to small bowel manifestations, including inflammation, mucosal injury and microthrombi formation [23].

**Figure 5:**
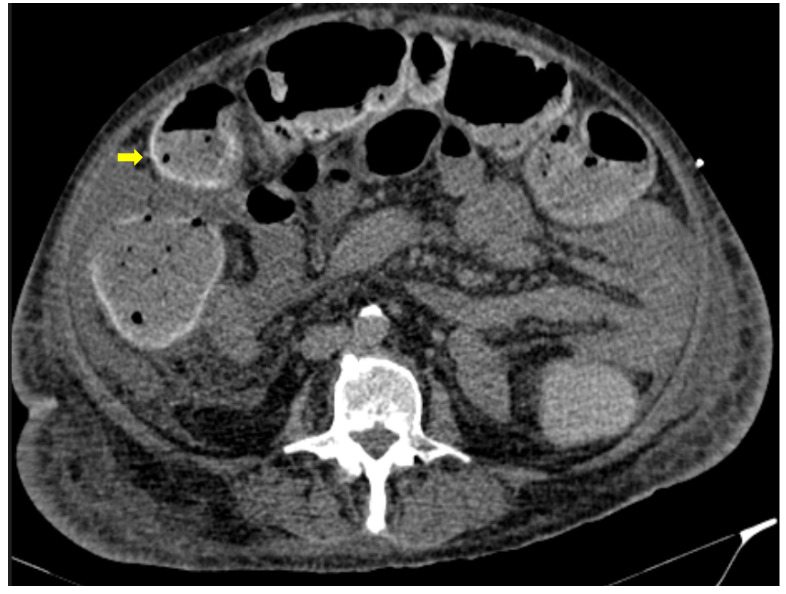
59-year-old female patient with severe COVID-19 pneumonia hospitalized in the ICU. CT demonstrating distended large bowel with hyperenhanced walls and free abdominal fluid (shock bowel)—yellow arrow. Note the concomitant subcutaneous edema.

Several studies have investigated the CT findings associated with small intestine involvement. In the CT scans of 149 COVID-19 patients, nearly 20% were indicative of small intestine involvement, with wall thickening, luminal dilatation and mucosal enhancement, especially in severe cases associated with worse outcomes (Figure 5) [22].

**Figure 6:**
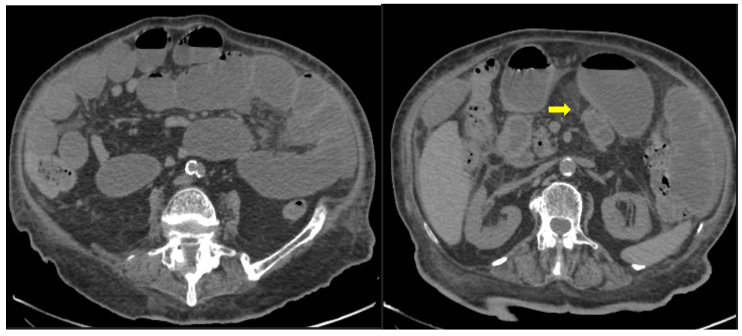
85-year old-patient with small bowel ileus (fluid filled loops—yellow arrow) at the 4th day of hospitalization.

Bhayana et al. found that 29% of CTs showed bowel wall thickening involving the colon or small bowel, such as findings of ischemia with pneumatosis or portal venous gas and bowel perforation and a fluid-filled colon in 43% of patients, suggestive of diarrhea. In addition to small bowel abnormalities, (Figure 6) CT imaging has also shown evidence of mesenteric lymphadenopathy, with mesenteric lymph node enlargement and increased enhancement in 22% of patients [9].

However, as these findings are not specific, CT findings must be correlated with clinical and laboratory data to confirm a COVID-19 diagnosis.

### 2.3. Hepato-Biliary Involvement

ACE2 receptors are found in many organs including the liver and biliary system, thus giving the opportunity for a local inflammation of these organs [24].

The hepatobiliary involvement in patients with COVID-19 infection and the abdominal imaging findings in those patients represent an interesting topic for further investigation.

One of the most common imaging findings in CT examinations of COVID-19 patients with hepatobiliary dysfunction (both in the literature and in our series) is hepatomegaly with or without a diffuse decrease in liver/spleen attenuation ratio. This can be due to liver injury due to the virus, pre-existing comorbidities (obesity, nonalcoholic fatty liver, and hepatitis) or as a result of hospitalization (parenteral nutrition and hepatotoxic drugs) [25,26].

In severe cases of COVID-19 infection, mostly in patients in ICU, a heterogenicity and mosaic pattern of the liver parenchyma is seen, combined with periportal edema and a contrast reflux in dilated hepatic veins indicating liver congestion due to hypoxia and cardiac failure [25].

**Figure 7:**
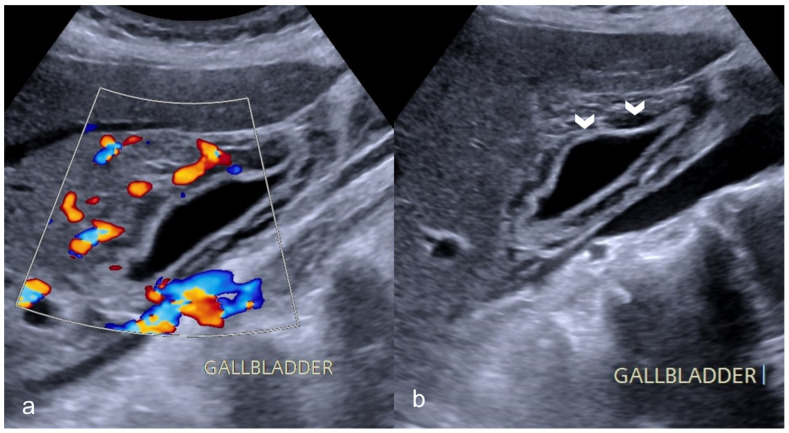
A 69-year-old man with COVID-19 presented a positive Murphy sign. Ultrasound images showed gallbladder wall striation and increased thickness ((**b**)-arrowheads). The Color Doppler technique revealed hyperemia (**a**). No gallbladder stones were present indicating acalculous cholecystitis.

**Figure 8:**
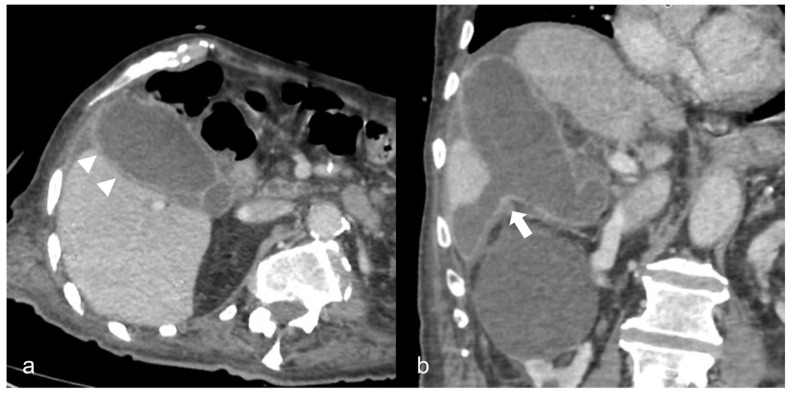
A 90-year-old COVID-19 patient presented acute right quadrant abdominal pain and cholestasis. Computed tomography on the axial (**a**) and coronal (**b**) plane showed acute cholecystitis with diffuse thickening of the gallbladder wall and pericholecystic fluid (arrowheads). Disruption of the gallbladder wall (arrow) is also present with fluid collection around the liver indicating perforation.

Biliary imaging findings are also encountered in patients with COVID-19 infection. In our institution, many patients presented with right quadrant pain and nausea/vomiting after a meal. In ultrasound and CT examinations, the findings included gallbladder distention, wall thickening and mural edema, pericholecystic fluid and inflammatory fat stranding with calculi and/or sludge (Figure 7 and Figure 8).

Less frequently, acalculous cholecystitis with sludge is seen in patients in the ICU, indicating cholestasis due to parenteral nutrition and/ or systemic inflammation [27,28].

### 2.4. Pancreatic Involvement

The data on pancreatic involvement during SARS-CoV-19 infection are limited. The frequency and severity of pancreatic damage and acute pancreatitis (AP) and its pathophysiology are still being studied.

AP is usually caused by increased alcohol consumption and gallstones. However, in 10%–20% of cases, an etiological factor cannot be identified [29]. A number of infectious agents, such as Coxsackie B virus and hepatitis A virus, infect the pancreas [30]. Radiology and imaging findings play a vital role mainly in the detection and follow up of complications of AP [30].

According to the revised Atlanta classification, the diagnosis of AP requires two of the following: (a) typical abdominal pain, (b) a serum lipase level (or amylase) at least three times greater than the upper normal limit and (c) characteristic imaging findings on CT, MRI or ultrasonography [31].

A study performed in pigeons with severe pancreatitis has managed to isolate COVID-19 or a Coronavirus-like virus, but no similar studies have been conducted in humans [32]. SARS-COV-19 uses ACE-2 receptors to enter pancreatic ductal cells [33], a fact that could explain the infection of the gland. Another possible way to induce the pancreatic injury is the caused cytokine storm, which produces pancreatic inflammation and an uncontrolled inflammatory immune systemic response caused by COVID-19. Finally, another important cause is the drug-induced pancreatic injury from antivirals, non-steroidal anti-inflammatory drugs (NSAIDs), tocilizumab and baricitinib, which belong to the approved treatment of COVID-19 [4].

Amylase and lipase elevation has been reported in 8.5–17.3% of patients with COVID-19. However, those enzymes are also increased in other gastrointestinal diseases, such as gastritis and colitis that have also been reported in COVID-19 patients. A meta-analysis of patients with COVID-19 showed that 18% had gastrointestinal symptoms and raised pancreatic enzymes could not be directly associated with pancreatitis [34]. Furthermore, kidneys play a major role in the clearance of both amylase and lipase, and their insufficiency could result in the elevation of these enzymes [35]. It has been proved that COVID-19 can infect insulin-producing cells in the pancreas and change their function, potentially explaining the high privilege of diabetes in previously healthy individuals [36].

**Figure 9:**
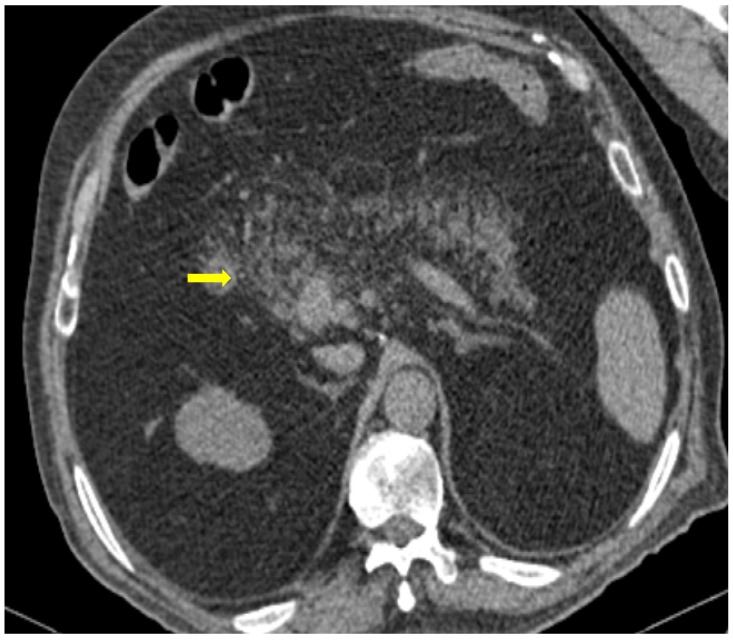
81-year-old patient with COVID-19 pneumonia developed epigastric pain at the second day of hospitalization. CT demonstrating acute pancreatitis (yellow arrow).

The temporal relationship between the onset of COVID-19 infection and inflammation of the pancreas has not been clearly established. Some patients develop COVID-19 symptoms and abdominal pain when the infection begins, whereas others present with AP several days after COVID-19 diagnosis (Figure 9) [37].

A study of 52 patients with COVID-19 pneumonia showed that there was a 17% incidence of pancreatic injury. Kumar V. et al. studied patients with acute pancreatitis and COVID-19 infection and found that half of them developed AP after a median of 22.5 days from the onset of respiratory symptoms, while the rest of them were admitted for abdominal pain [38] Another systematic review, including overall 37 patients, summarized that AP might be the first symptom of COVID-19 [39]. In addition, COVID-19 may negatively influence the morbidity and mortality linked with AP [40].

Finally, it should be noted that after the COVID-19 pandemic, pancreatic cancer and metastases rates have been dramatically raised, as there was a temporary cessation of screening during the pandemic. Less than 25% of patients had regular availability of diagnostic and staging tools, while 20% were unable to perform surgery [41].

### 2.5. Thromboembolic Complications

Systemic coagulopathy is common in COVID-19 patients with severe pneumonia [42].

Despite the limited available data, many thrombotic manifestations regarding the abdominal vessels have been documented in cases of COVID-19 inpatients. The CT depiction frequency is highly dependent on the performed protocol, which is usually unenhanced or contains only a portal venous phase. Only in a few cases the image acquisition is accomplished through an abdominal multiphase CT angiogram. Consequently, thromboembolic events regarding the arterial abdominal branches are scarcely reported, opposed to venous thrombi, possibly due to underdiagnosis [43].

In many cases we can only depict indirect findings due to the small vessel thrombotic nature of the disease, a phenomenon that could also be related to hospitalization or comorbidities [11]. When it comes to microvasculature, both arteries and veins are affected [44]. An interesting fact is that in an accountable percentage of the arterial thrombi, the affected vessels did not have any atherosclerotic alterations, suggesting that COVID-19 was the generating factor of the thrombus [45].

Solid abdominal organs infarcts have been documented during imaging protocols performed for pulmonary embolism detection, especially in patients with elevation of the D-dimers. Apart from those cases, renal infarcts were reported in scans performed for vague abdominal pain or due to acute kidney failure [45].

Fewer reports about splenic infracts in COVID-19 patients also come from thoracic scans with abdominal extension [46].

**Figure 10:**
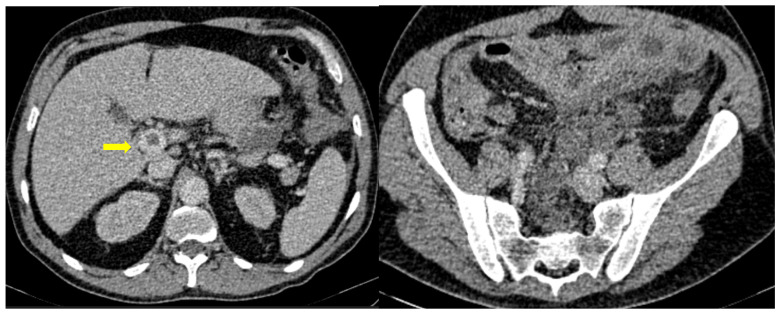
56-year-old hospitalized male patient with COVID-19 pneumonia with raised level of d-dimers. CT depicting the thrombosed portal vein (yellow arrow). Note that in the same scan, there is extended small bowel thickening with mesenteric free fluid.

A single-center small retrospective study reported in COVID-19 patients 15 cases of acute aortic thrombosis, splenic artery thrombosis (associated with splenic infraction), superior mesenteric and renal artery thrombosis such as a celiac and an internal iliac thrombosis. An interesting part was a patient with infrarenal aortic wall inflammation and focal dissection, while many venous thromboses have been described (affecting the portal vein, inferior, superior mesenteric, renal, ovarian vein and inferior vena cava). Reports of indirect findings of the splanchnic branch venous occlusion described bowel wall severe edema, hyperenhancement or severe hypoenhancement, associated mesenteric and portal intravenous gas, bowel pneumatosis and pneumoperitoneum (Figure 10) [47]. Bari Dane et al. published a case of a simultaneous nonocclusive aortic, celiac and superior mesenteric artery thrombus combined with a complete common hepatic artery thrombus [48].

Those findings are in accordance with reports of bowel pneumatosis as a thrombotic event outcome [49], while many case reports and large case series demonstrate major abdominal—both arterial and venous—thrombosis in COVID-19 patients. In fact, many patients suffer from thrombotic occlusion despite prophylaxis or even the full-dose anticoagulation therapy supporting evidence of COVID-19 direct endothelial injury [50].

### 2.6. Bleeding Manifestations

Bleeding in patients with COVID-19 can be the result of pre-existing risks factors, antithrombotic drugs and a massive immune response to the virus, [51]. A common bleeding complication is abdominal hematomas, usually of the Iliopsoas and rectus abdominis muscle [51,52]. The role of a CT scan is major in both the diagnosis and treatment of these entities. They are usually seen as muscle enlargement with increased densities, blood-fluid level and possibly extravasation of contrast. When it comes to the GI tract, upper GI is the most common site of bleeding followed by the lower GI [53,54].

A potential pathogenic route is through the binding of the virus with Angiotensin Converting Enzyme-2 expressed in gastrointestinal epithelial cells [55].

Underlying mucosal lesions in the GI (ulcers and vascular abnormalities) and prophylactic/therapeutic anticoagulant therapy should be considered and further investigated.

**Figure 11:**
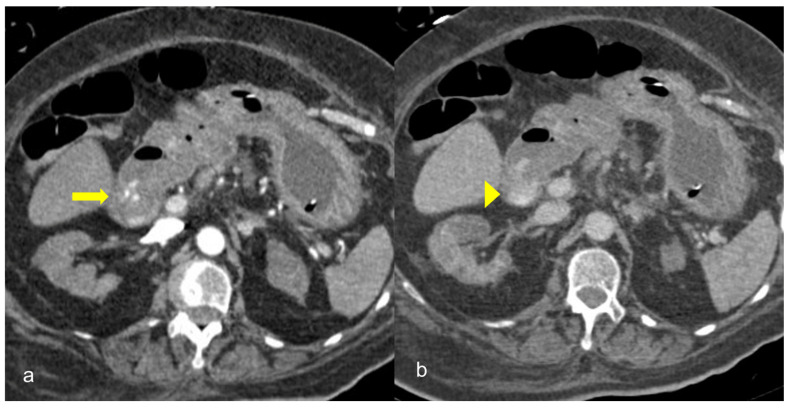
A 75-year-old intubated patient with COVID-19 presented with hematemesis, hypotension and acute drop of hemoglobin level. Computed Tomography revealed active intraluminal extravasation of contrast into the 2nd part of duodenum (arrow) on arterial phase (**a**). Further pooling of the contrast (arrowhead) is shown on portal phase (**b**).

GI hemorrhage is less commonly encountered in abdominal imaging, and CT findings include active intraluminal extravasation of contrast and indirect signs such as luminal distention with blood clots (Figure 11) [56].

Imaging is additionally significant to the treatment plan by identifying the exact site and extent of the bleeding as well as offering a precise and minimally invasive treatment option. Digital Subtraction Angiography can confirm the active bleeding seen as a “contrast blush”, detect the responsible branch and provide occlusion via selective embolization.

## 3. Results

In our institution, 84 COVID-19 patients underwent abdominal CT imaging studies displaying positive GI imaging findings. The study population included 43 males and 41 females. The oldest patient was 90 years old, and the youngest patient was 18 years old. Fourteen patients were admitted to the ICU, and in total 26 patients died.

The most frequent indications for abdominal CT were abdominal pain and abdominal distention. The majority of the patients presented elevated levels of liver enzymes and C reactive protein.

The most frequent comorbidities included hypertension, heart disease, dyslipidemia, diabetes mellitus, obesity and cancer. Also, a patient with kidney transplant was included.

The most common imaging findings concerned the small intestine and colon. Bowel wall thickening was noted in 25 patients (22 of them in colorectal area and the rest in small bowel) accompanied with pericolic fluid (6 patients), submucosal edema (6 patients) or intestinal perforation (2 patients). Furthermore, thrombosis of SMA was noted in one patient.

Hepatobiliary manifestations were also common with 22 patients presenting liver steatosis, 3 patients suffering from acute cholecystitis and 24 patients in total presenting biliary sludge and/or gallstones.

Seven patients experienced pancreatitis during their hospitalization.

In our institution, we are in concordance with the few publications about the abdominal manifestations of COVID-19. It was not our intention to present a statistical analysis of our findings but to expound upon the plethora of imaging findings in regard to the digestive system involvement.

In the future, a more extensive study and analysis should take place, in collaboration with other tertiary referral centers, using a bigger sample.

## 4. Conclusions

COVID-19 is a multi-organ disease, and GI manifestation might be noted at the time of diagnosis or later in the course of the disease. The presence of GI manifestations is associated with increased clinical severity and poor outcome. Therefore, it is pivotal for radiologists to be aware of related imaging findings to aid diagnosis and appropriate management.

## Data Availability

Not available due to confidentiality of imaging examinations.
